# Variation in candidate genes *CLOCK* and *ADCYAP1* does not consistently predict differences in migratory behavior in the songbird genus *Junco*

**DOI:** 10.12688/f1000research.2-115.v1

**Published:** 2013-04-22

**Authors:** Mark P Peterson, Mikus Abolins-Abols, Jonathan W Atwell, Rebecca J Rice, Borja Milá, Ellen D Ketterson

**Affiliations:** 1 Department of Biology and Center for the Integrative Study of Animal Behavior, Indiana University, Bloomington IN, 47408, USA; 2 National Museum of Natural Sciences, Spanish Research Council (CSIC), Madrid, 28006, Spain

## Abstract

Recent studies exploring the molecular genetic basis for migratory variation in animals have identified polymorphisms in two genes ( *CLOCK *and ADCYAP1) that are linked to circadian rhythms and correlate with migratory propensity and phenology among individuals and populations. Results from these initial studies are mixed, however, and additional data are needed to assess the generality and diversity of the molecular mechanisms that regulate the biology of migration. We sequenced CLOCK and ADCYAP1 in 15 populations across the two species of the avian genus *Junco*, a North American lineage in which multiple recently diverged subspecies and populations range from sedentary to long-distance migrants. We found no consistent associations between allele length and migratory status across the genus for either CLOCK or ADCYAP1. However, within two subspecies groups, populations that migrate longer distances have longer CLOCK alleles on average. Additionally, there was a positive relationship between ADCYAP1 allele length and migratory restlessness (zugunruhe) among individuals within one of two captive populations studied—a result similar to those reported previously within captive blackcaps ( *Sylvia atricapilla*). We conclude that, while both ADCYAP1 and CLOCK may correlate with migratory propensity within or among certain populations or species, previously identified relationships between migratory behavior and sequence variants cannot be easily generalized across taxa.

## Introduction

Every year billions of birds make round-trip flights from their breeding grounds to suitable winter climes and back again ^1^. This seasonal migration requires immense coordination of different systems including fattening, locomotion, orientation, and activity ^2^. Several aspects of migratory behavior including onset, duration, intensity, and orientation are known to be heritable and to respond quickly to artificial and natural selection – indicating a degree of genetic control ^3– 7^. However, other studies indicate that both learning and developmental environments can also shape migratory phenotypes ^2, 8, 9^.

The propensity to migrate appears extremely labile on an evolutionary scale, with shifts between migratory and sedentary status independently arising, often repeatedly, in multiple lineages ^10– 12^. In recent decades, many populations and species are reducing the distance or frequency of migrations, and others are ceasing to migrate altogether, presumably in response to changing environments world-wide ^7, 13– 15^. Understanding the molecular genetic mechanisms that regulate migration biology is imperative both for 1) understanding the evolutionary processes of behavioral adaptation and diversification, and 2) conserving and managing the phenomenon of animal migrations in the face of ongoing environmental change. However, relatively few studies have addressed the molecular genetic mechanisms of migration ^16^. Our goal in this study was to build upon previous candidate gene studies to determine whether or not similar genetic mechanisms explain variation in migratory propensity at the level of individuals, populations, and species across taxa.

Previous studies have identified a number of candidate genes that may be involved in regulating migration, with particular focus on circadian rhythm genes. For example, the gene *CLOCK* controls several aspects of the circadian rhythm in mice ^17^, and in the blue tit ( *Cyanistes caeruleus*) it varies across latitudinal clines ^18^, and with timing of breeding ^19^. In salmon ( *Oncorhynchus* spp.), *CLOCK* microsatellite repeat length also varies geographically and predicts migratory timing ^20– 22^. Monarch butterflies ( *Danaus plexippus*) use *CLOCK* as part of the circadian pathway that guides their annual migration ^23^, suggesting that *CLOCK* may play a role in the timing of migration across vertebrate and invertebrate lineages. However, blackcaps ( *Sylvia atricapilla* ^24^) bluethroats ( *Luscinia svecica* ^18^), and swallows of the genus *Tachycineta* ^25^ do not show such correlations between this *CLOCK* polymorphism and migratory behavior or geographic location, despite possessing genetic variation at the locus. Furthermore, barn swallows ( *Hirundo rustica*) show substantial variation in migratory phenotype, but very little variation at this *CLOCK* polymorphism ^26^.

In another gene, adenylate cyclase-activating polypeptide 1 ( *ADCYAP1*), length of a microsatellite repeat predicts migratory propensity of both populations and individuals in blackcaps ^24^. *ADCYAP1* is expressed throughout the brain and body of vertebrates ^27^ and encodes pituitary adenylate cyclase-activating polypeptide (PACAP) (reviewed in ^28^). PACAP is involved in many diverse behavioral and physiological phenotypes, including the circadian system (reviewed by Vaudry *et al.* ^28^). Specifically, in chickens ( *Gallus gallus*), PACAP directly activates *CLOCK* and other circadian genes in the pineal gland ^29^. Further, PACAP stimulates release of melatonin ^30^, and is a key component in entraining the circadian system to the light-dark cycle ^31^, suggesting that it may be involved in circannual rhythms that rely on sensing day length, such as migration. Additionally, PACAP is involved in a large number of physiological and behavioral effects, from feeding behavior to breathing ^28^, many of which may be related to physiological changes involved in migration ^24^.

The body of work to date thus indicates that certain genes are associated with migratory phenotype in some way across phyla and within classes. However, these findings also warrant further study of the genetics of this complex phenotype at multiple levels in independent systems to determine whether selection is using conserved genetic machinery to arrive at similar phenotypic outcomes (e.g., Korsten *et al.* ^32^).

Here, we examined allelic variation in both *CLOCK* and *ADCYAP1* in the genus *Junco* at the levels of species, populations, and individuals. The junco species group is currently comprised of the yellow-eyed junco ( *J. phaeonotus*) and the dark-eyed junco ( *J. hyemalis*), each of which contains multiple subspecies and populations showing a wide range of migratory behaviors ^33– 35^. Several of these subspecies have been identified as distinct species in the past (e.g. Miller ^35^) and investigations into the genetic structure and systematics of the group are ongoing (e.g. Milá *et al.* ^36^, McCormack *et al.* ^37^, Rasner *et al.* ^38^ and Whittaker *et al.* ^39^).

The various dark-eyed junco subspecies offer an especially exciting opportunity to investigate migratory differences between closely related populations. There are 15 subspecies comprising the species ^34^, and genetic similarity indicates that these closely related forms may have radiated just within the last 10,000 years–spreading across North America following the most recent glacial maximum ^36^. This rapid diversification has limited the ability to identify clear phylogenetic patterns within the species ^36^. These subspecies and populations differ markedly in migratory phenotype, ranging from completely sedentary to those that migrate thousands of kilometers ( Table 1), with several populations also exhibiting individual variation in migratory behavior in which some individuals migrate and others do not ^12, 34, 35, 40^. Further, a coastal population of the Oregon junco ( *J. h. thurberi*) has diverged from a montane altitudinal migrant population in the last 30 years and become sedentary in a milder environment on the campus of the University of California in San Diego ^41, 42^.

**Table 1.** Sampled populations and assigned migratory scores. Group categories follow generally from distinct groups of subspecies or unique forms as discussed by Milá *et al.* (2007) ^36^ or Nolan *et al.* (2002) ^34^. Subspecies designations follow Miller (1941) ^35^ as summarized by Nolan *et al.* (2002) ^34^ and Sullivan *et al.* (1999) ^33^; dark-eyed subspecies are noted as *J. h.* spp. and yellow-eyed subspecies as *J. p*. spp. Migratory Behavior codes indicate the range of migratory behaviors inferred from breeding and wintering distributions, as follows: **LD2** = long distance II, at minimum 1600 km up to 5600 km, depending on migratory connectivity; **LD1** = long distance I, likely 400–700 km, possibly up to 5000 km, depending on migratory connectivity; **R** = regional, typically greater than 200 km; **A** = altitudinal, typically less than 200 km; **P** = apparent partial migration documented; **F** = apparent facultative migration documented; **S** = sedentary (see text for references). Migratory score ordinally ranks population migratory propensity, similar to previous published accounts ^24^.

Group	Subspecies	Sampling site	Range	Abbreviation	Migratory behaviors	Migratory score
Slate-colored	*J. h. hyemalis*	Mississippi, USA	Wintering	SCJU-MS	LD2	6
Slate-colored	*J. h. hyemalis*	Indiana, USA	Wintering	SCJU-IN	LD1	5
Slate-colored	*J. h. hyemalis*	Michigan, USA	Wintering	SCJU-MI	R to LD	4
Carolina	*J. h. carolinensis*	Virginia, USA	Breeding	CRJU	A, P, F	2
White-winged	*J. h. aikeni*	South Dakota, USA	Breeding	WWJU	R to LD1; A, P, F	3
Oregon	*J. h. oreganus*	British Columbia, Canada	Breeding	ORJU-BC	R to LD1; P, F	3
Oregon	*J. h. thurberi*	Mt. Laguna, California, USA	Breeding	ORJU-LM	A, P, F	2
Oregon	*J. h. thurberi*	San Diego, California, USA	Year-round	ORJU-SD	S	1
Gray-headed	*J. h. caniceps*	Utah, USA	Year-round	GHJU	R	3.5
Pink-sided	*J. h. mearnsi*	Wyoming, USA	Breeding	PSJU	R to LD1	4.5
Guadalupe	*J. h. insularis*	Guadalupe Island, Mexico	Year-round	GUJU	S	1
Yellow-eyed	*J. p. phaeonotus*	Durango, Mexico	Year-round	YEJU-DO	S	1
Yellow-eyed	*J. p. phaeonotus*	Mexico City, Mexico	Year-round	YEJU-DF	S	1
Guatemala	*J. p. alticola*	Huehuetenango, Guatemala	Year-round	GTJU	S	1
Baja	*J. p. bairdi*	Baja California Sur, Mexico	Year-round	BAJU	S	1

The diversity of migratory behavior along with the close genetic relationships among divergent subspecies and populations make the junco species an excellent system in which to further examine the role of candidate genes linked to migration. Based on earlier studies of *CLOCK* and *ADCYAP1* variation ^18, 24, 43^, we predicted that junco populations that migrate longer distances would possess longer microsatellite repeat-length alleles of both *CLOCK* and *ADCYAP1* when compared with those that migrate shorter distances including altitudinal migrants and sedentary populations. We also predicted that allele length would positively covary with individual variation in migratory restlessness within populations.

## Methods

### Classifying population migratory behavior

Populations of juncos were classified according to a scale of migratory distance based on published reports in the literature ^33– 35, 40– 42, 44, 45^, personal observation. Each population was ranked on a scale from 1 (sedentary) to 6 (long-distance migrant) based on the average distance migrated by individuals in that population ( Table 1). Distance migrated also varies within populations. For example, in the Eastern United States, females ^46^ and adults ^47^ generally migrate farther from their breeding ranges than males and yearlings, respectively. Some junco populations also exhibit partial migration, in which a subset of individuals remains on the breeding grounds while others depart ^34, 35^. The classification system employed here broadly categorizes the general migratory phenotype of each population and was organized to resemble previous reports for other species (e.g. blackcaps ^24^).

### Assessing the migratory behavior of individuals

Individuals from two populations of *J. h. thurberi* residing in southern California were captured as recently independent offspring and held in captivity under identical conditions in a ‘common garden’. Individuals were held individually (0.61 m × 0.61 m × 0.61 m cages) in a climate controlled indoor aviary with *ad libitum* access to food and water. Individuals were neither visually nor acoustically isolated, and light regimes were changed bi-weekly to match photoperiod at their home latitude. All methods were reviewed and approved by the IACUC at Indiana University (protocol #09-037). To reduce the probability of capturing siblings and pseudoreplication, individuals were captured from multiple locations, and one individual from each identified sibship (one in each population) was randomly included in all analyses, as in previous studies ^24^. Additional studies of these captive common garden populations are reported elsewhere ( ^48^; Atwell *et al.* in review).

Briefly, spring nocturnal restlessness behavior was scored from 3 March to 1 August 2010 by recording intervals of night-time perch-hopping activity of birds housed indoors in individual cages equipped with microswitched perches. This method is considered effective to capture 95% of migratory behavior observed in infrared video and ultrasound methods ^49^, and even non-traditional songbird migrations tend to occur at night ^50, 51^. Juncos typically migrate at night ^34^, and this sampling period overlapped with the typical spring migration as well as breeding seasons of dark-eyed juncos ^34, 41^. As was found in prior studies of migratory restlessness in the junco (e.g., Ketterson and Nolan ^52^) and other species, migratory restlessness extended into the typical breeding period, perhaps due to caged birds’ inability to perform reproductive activities ^53^. Nocturnal hops were recorded starting 30 minutes after dusk until 30 minutes before dawn under a daylight regime that simulated the photoperiod at their native latitude. GraphPad Prism 3.0 (GraphPad, USA) was used to quantify the seasonal intensity of individual spring migratory restlessness by calculating the area under each fitted seasonal profile curve, which provided a total migratory propensity score for each bird (n = 36 ^14^; Atwell *et al.* in review).

### Sampling

Analysis of population differences in genotype was conducted using DNA collected between 1996–2010 from adult juncos that were captured from breeding or wintering populations as a part of other studies. Less than 100 μL of whole blood was collected via capilary tube from a small puncture in the alar vein for future extraction. Both males and females were included, and in all populations in which sexes were known, the sexes did not differ in allele length for either locus (t-test, all p > 0.1). Birds sampled in their winter range were captured from December through February, after juncos are thought to have arrived at their wintering grounds and before they have departed in the spring ^34, 54^. All individuals from breeding locations were captured as adults during the breeding season of that population. No known siblings were included in these analyses.

### Genotyping

We extracted DNA from whole blood from 15 populations spanning the range of migratory phenotypes in the genus *Junco* ( Table 1). We developed primers surrounding a microsatellite repeat of interest based on previously published reports for *CLOCK* ^18, 43^ and *ADCYAP1* ^24, 43^ in birds, and we modified the primers to more closely match the junco sequence based on the junco transcriptome ( ^55^; See Table 2 for primer details). The primers were used to amplify the respective polymorphisms in a single multiplex reaction using an amplification kit and following the manufacturer’s directions (Qiagen Inc, Valencia, California, USA). An initial 15´ heat activation cycle (95°C) was followed by 35 cycles of 94°C denaturation for 30”, 60°C annealing for 90”, and 72°C extension for 60”, and a final elongation of 10´. PCR products were then diluted 1:200 in ddH _2_0 with LIZladder (Applied Biosystems, Carlsbad, California, USA), denatured at 95°C for 5´, and placed on ice. Samples were analyzed on an ABI 3730 using Peak Scanner v1.0 (Applied Biosystems, Carlsbad, California, USA).

**Table 2.** Primers used in this study. Both primers are based on Steinmeyer *et al.* ^43^, and *CLOCK* was modified to match junco sequences ^55^.

Gene name	Forward primer 5´ → 3´	Reverse primer 5´→ 3´
***CLOCK***	TTTTCTCAAGGTCAGCAACTTGT	CTGTAGGAACTGCTGGGGKTGCTG
***ADCYAP1***	GATGTGAGTAACCAGCCACT	ATAACACAGGAGCGGTGA

### Data analysis

In order to assess the relationship between allele length and migratory behavior among populations for both loci, we performed Spearman's correlations between population mean allele length and population migratory status. Qualitatively similar results are obtained with major allele count methods that have been applied in previous studies ^18, 24^ and when only dark-eyed subspecies were considered (data not shown). Within the sub-species groups for which we sampled multiple populations (Oregon juncos and slate-colored juncos, see Table 1), we additionally performed a nested ANOVA on allele lengths with population as a grouping factor within each subspecies to determine whether mean allele length differed significantly between populations or these subspecies.

To relate individual variation in allele length of *ADCYAP1* and *CLOCK* to migratory restlessness, we fit linear models with migratory restlessness as the dependent variable and all combinations of population, mean allele length of the individual, sex, and each pairwise interaction as predictors. The resulting models were compared against each other using Akaike's Information Criterion. When the best fit model yielded a significant interaction term, we followed with a separate linear model for each population ^56^.

All statistical analyses were performed in R version 2.15.2 ^57^. Hardy-Weinberg equilibrium and heterozygosity were calculated with the package genetics ^58^. All reported p-values are two-tailed, and hence conservative with respect to *a priori* directional hypotheses.

## Results

### Among population comparisons for *ADCYAP1*

We identified 16 alleles at the *ADCYAP1* locus ranging in length from 154 to 181 base pairs (bp), and all populations contained between five and ten alleles ( Table 3). Allele lengths of 161 or 163 bp were most common in all populations. Nearly all of the identified alleles differed by multiples of two bases from these common alleles. The exceptions were represented in only 12 individuals, including five individuals from the Laguna Mountain population that shared an allele 164 bp long. All populations, both separately and combined, were in Hardy-Weinberg equilibrium (all p > 0.73).

**Table 3.** Distribution of *ADCYAP1* allele length within each population. Table lists the number of individuals from each population possessing each of the identified *ADCYAP1* alleles. Number of individuals genotyped is indicated as “n” for each population. Het = calculated heterozygosity for the population. Other abbreviations follow from Table 1.

Population	154	155	156	157	158	159	160	161	162	163	164	165	167	169	171	181	n	Het
**SCJU-IN**	1	3	0	3	0	9	0	11	0	8	0	8	1	1	1	0	19	0.809
**SCJU-MI**	0	1	0	1	0	11	1	11	2	17	0	11	2	1	0	0	23	0.852
**SCJU-MS**	0	0	0	4	0	6	0	8	0	13	0	4	2	1	0	0	29	0.816
**CRJU**	0	0	0	2	0	9	0	19	0	12	0	1	1	0	0	0	22	0.710
**WWJU**	0	0	1	5	0	1	0	35	0	7	0	5	1	1	0	0	28	0.587
**ORJU-BC**	0	1	0	1	0	3	0	12	0	8	0	2	3	0	0	0	15	0.768
**ORJU-LM**	0	0	0	1	0	11	0	18	0	16	5	14	1	0	0	0	32	0.802
**ORJU-SD**	0	0	0	10	0	8	0	26	0	15	0	3	12	0	0	0	36	0.791
**GHJU**	0	0	0	2	0	5	1	17	0	17	0	8	4	0	0	0	27	0.778
**PSJU**	0	1	0	4	0	2	0	16	0	14	0	7	2	2	0	0	24	0.786
**GUJU**	0	0	0	0	0	6	0	11	0	6	0	8	4	0	0	1	18	0.811
**YEJU-DO**	0	3	0	2	0	4	0	10	0	1	0	0	6	2	0	0	14	0.812
**YEJU-DF**	0	0	0	2	1	18	1	17	0	13	0	4	6	0	0	0	31	0.794
**GTJU**	0	0	0	4	0	8	0	13	0	0	0	7	2	0	0	0	17	0.761
**BAJU**	0	0	0	0	0	2	0	3	0	21	0	16	3	1	0	2	24	0.700

**Figure 1.**
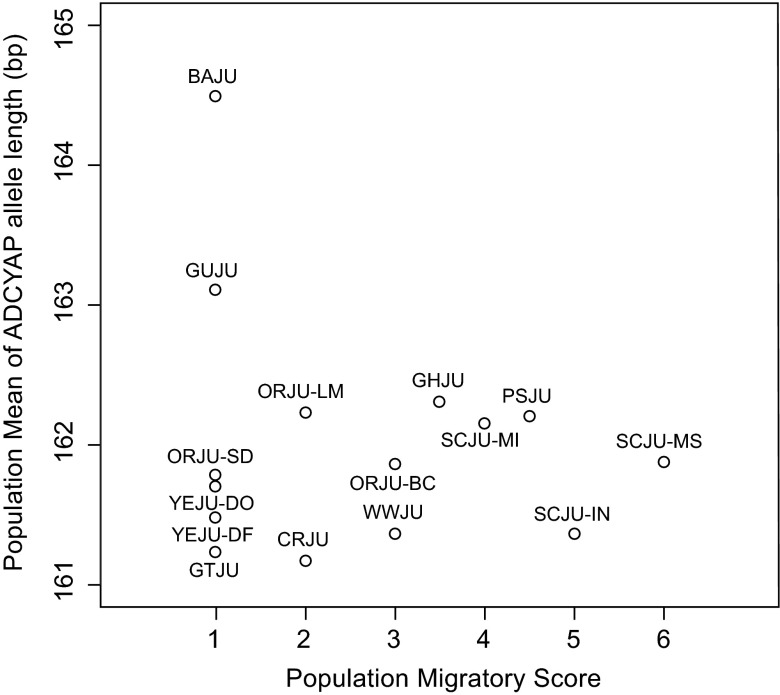
*ADCYAP1* allele length and migratory status. No correlation was found between allele length and migratory score (Spearman's correlation, p > 0.05).

**Figure 2.**
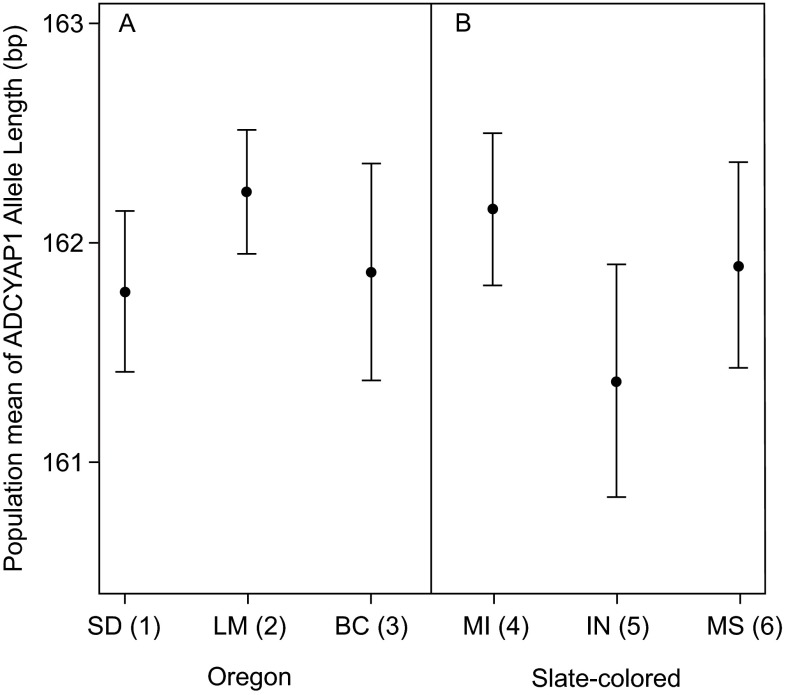
*ADCYAP1* allele length within sub-species groups. *ADCYAP1* allele length did not differ between populations within the sub-species for which we have multiple sampling locations. Shown are means and standard errors for Oregon juncos listed by breeding grounds (panel A) and slate-colored juncos listed by wintering grounds (panel B). Population migration scores are shown in parentheses with higher numbers indicating populations that migrate longer distances (see Table 1 for more details).

There was no correlation between the population mean allele length of *ADCYAP1* and population migratory status (Spearman's correlation, rho = 0.022; p = 0.938; Figure 1). Within the sub-species groups sampled at multiple locations the length of the *ADCYAP1* allele did not differ between subspecies, or within subspecies (nested ANOVA, subspecies difference: F _1,296_ = 0.233, p = 0.629; population within subspecies: F _4,296_ = 0.852, p = 0.493; Figure 2).

### Among population comparisons for *CLOCK*

We identified eight alleles at the *CLOCK* locus that varied in length from 267 to 285 bp, and all but one population ( *J. h. insularis* from Guadalupe island) contained between two and four alleles ( Table 4). A single allele (276 bp) was the most common in all populations except *J. p. alticola* from Guatemala where the 279 bp allele was most common. All but one of the additional alleles (present in a single individual) differed from the most common allele by multiples of three base pairs. All populations, both separately and combined, were in Hardy-Weinberg equilibrium (all p > 0.05).

**Table 4.** Distribution of *CLOCK* allele length within each population. Table lists the number of individuals, from each population, possessing each of the identified *CLOCK* alleles. Number of individuals genotyped is indicated as “n” for each population. Het = calculated heterozygosity for the population. Other abbreviations follow from Table 1.

Population	267	269	270	273	276	279	285	n	Het
**SCJU-MS**	0	0	3	2	33	2	0	20	0.316
**SCJU-IN**	0	0	3	0	36	1	0	20	0.188
**SCJU-MI**	1	0	8	2	49	0	0	30	0.319
**CRJU**	0	0	9	0	36	0	1	23	0.357
**WWJU**	0	1	20	0	35	0	0	28	0.490
**ORJU-BC**	0	0	2	0	27	1	0	15	0.191
**ORJU-LM**	1	0	5	4	56	0	0	33	0.257
**ORJU-SD**	0	0	17	0	57	0	0	37	0.366
**GHJU**	0	0	3	1	52	0	0	28	0.137
**PSJU**	0	0	7	1	39	1	0	24	0.324
**GUJU**	0	0	0	0	36	0	0	18	0.000
**YEJU-DO**	0	0	3	2	24	5	0	17	0.483
**YEJU-DF**	0	0	5	0	53	4	0	31	0.263
**GTJU**	0	0	1	1	14	18	0	17	0.565
**BAJU**	1	0	1	2	44	0	0	24	0.160

**Figure 3.**
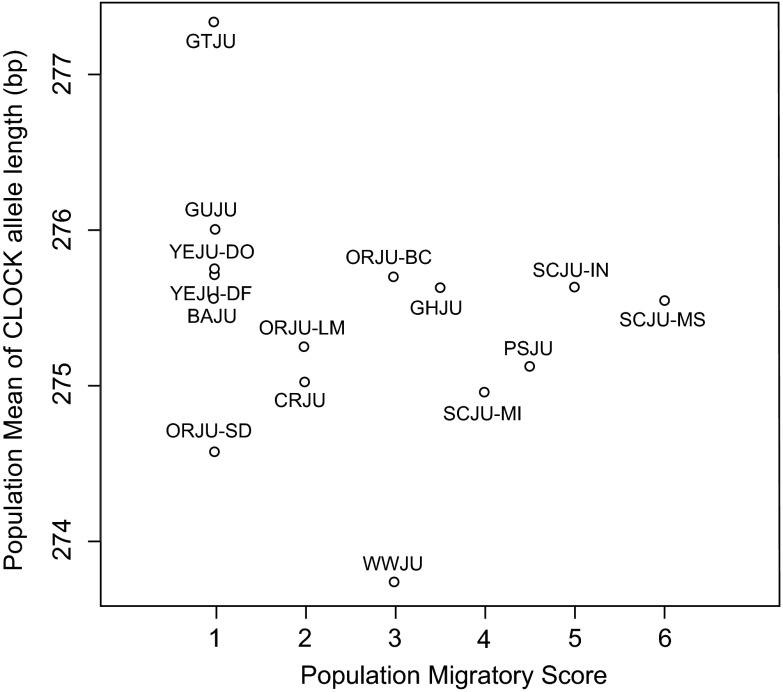
*CLOCK* allele length and migratory status. No correlation was found between population mean *CLOCK* allele length and migratory status (Spearman's correlation, p > 0.05).

**Figure 4.**
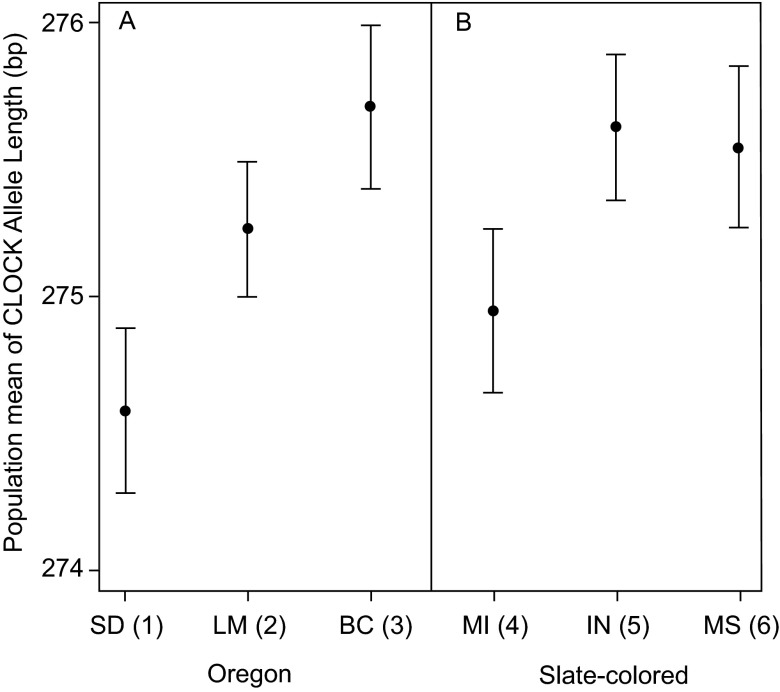
*CLOCK* allele length within sub-species groups. Within each subspecies group for which we have multiple sampling locations, there is a trend for populations that migrate a longer distance to have longer *CLOCK* alleles. Figure shows mean and standard error for Oregon juncos listed by breeding grounds (panel A; ANOVA, p = 0.056) and slate-colored juncos listed by wintering grounds (panel B; ANOVA, p = 0.18; see text for details). Population migration scores are shown in parentheses with higher numbers indicating populations that migrate longer distances (see Table 1 for more details).

Population mean allele length of *CLOCK* did not correlate with migratory status (Spearman's correlation, rho = -0.38, p = 0.159; Figure 3) when all populations were compared. Among the sampled populations of Oregon juncos ( *J. h. oreganus* and *J. h. thurberi*) and slate-colored juncos ( *J. h. hyemalis)* there was a significant effect of population within subspecies on *CLOCK* allele length, but not a difference in *CLOCK* allele length between populations (nested ANOVA, subspecies difference: F _1,300_ = 1.235, p = 0.267; population within subspecies: F _4,300_ = 2.468, p = 0.045; Figure 4). In both groups, populations that migrated longer distances possessed longer alleles at the *CLOCK* locus.

### Individual variation in migratory restlessness

**Figure 5.**
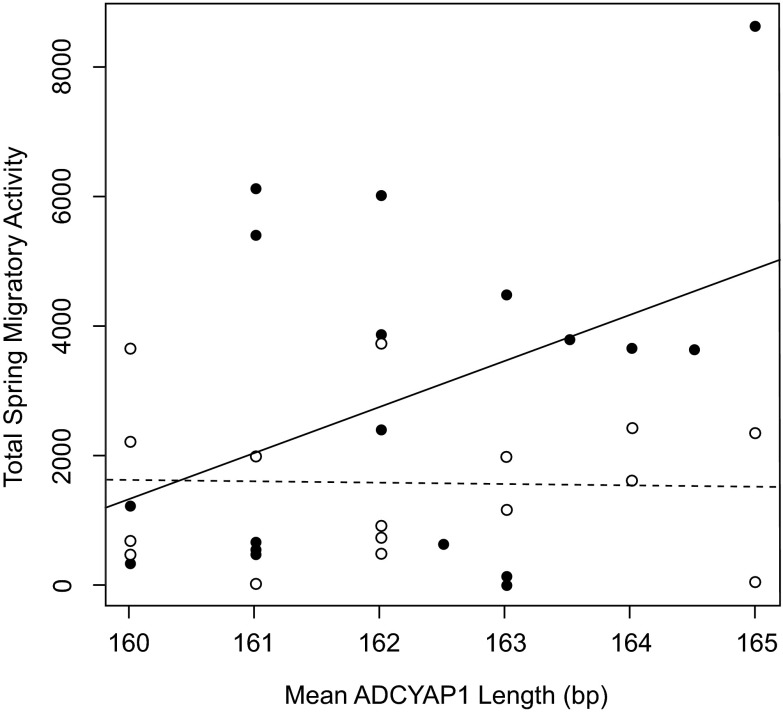
*ADCYAP1* and individual variation. Plot shows mean allele length of *ADCYAP1* against individual migratory restlessness score for Oregon juncos from the migratory population at Laguna Mountain, CA, USA (solid circles, solid line) and the sedentary population in San Diego, CA, USA (open circles, dashed line). Mean allele length marginally predicts migratory behavior in the Laguna Mountain, but not San Diego, populations.

We tested for an association between mean *ADCYAP1* allele length and level of migratory restlessness within the two captive populations of Oregon juncos ( *J. h. thurberi*, Laguna Mountain and UC-San Diego). The most informative linear model described individual migratory restlessness in relation to population, mean *ADCYAP1* allele length, and the population by allele length interaction (with Laguna Mountain as reference: effect of allele length = 712.9, p = 0.029; effect of population = 117,459.8, p = 0.056; population*allele length effect = -732.3, p = 0.053; model R ^2^ = 0.225, p = 0.036). This interaction model performed better than all other tested models (Akaike's Information Criterion = 669.3; all other models AIC > 670.8; see Methods for list of models). Because the interaction term indicates a different effect of allele length on migratory behavior in each population, we ran separate linear models for each population ^56^ which confirmed that longer individual mean *ADCYAP1* allele length marginally predicted greater migratory restlessness in the migratory Laguna Mountain population (effect of allele length = 712.9, R ^2^ = 0.169, p = 0.090; Figure 5, Supplementary Table 1), but not in the sedentary UC-San Diego population (effect of allele length = -19.36, R ^2^ = 0.001, p = 0.844; Figure 5).

**Figure 6.**
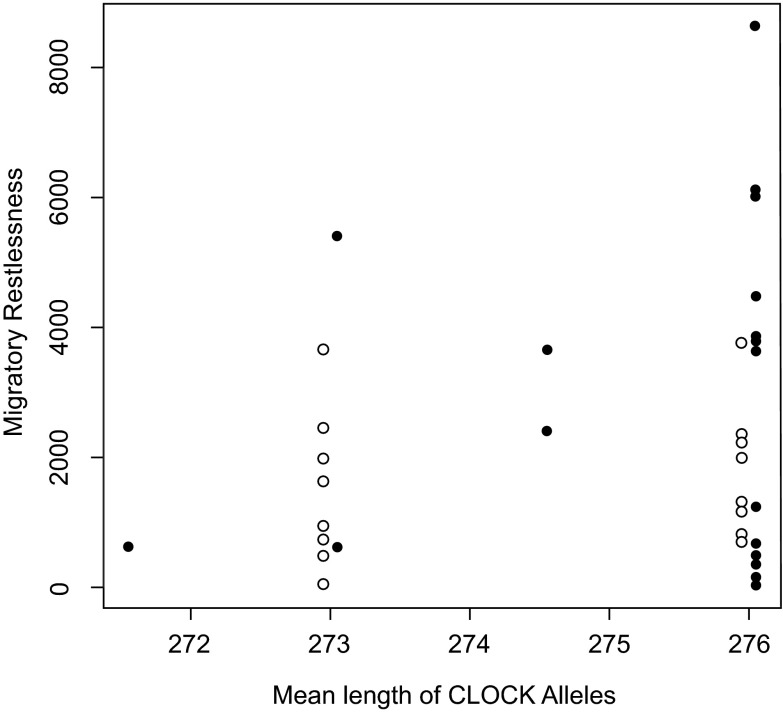
*CLOCK* and individual variation. Plot shows individual migratory restlessness score for Oregon juncos against the mean length of the *CLOCK* alleles from the migratory population at Laguna Mountain, CA, USA (solid circles) and the sedentary population in San Diego, CA, USA (open circles). Points are slightly jittered for each population to improve visualization. There is no significant relationship between *CLOCK* length and migratory restlessness.

There was no relationship between mean *CLOCK* allele length and individual migratory restlessness behavior ( Figure 6, Supplementary Table 1). The most informative linear model described only a population difference: the Laguna Mountain population displayed more restlessness than the population from UC-San Diego (with Laguna Mountain as reference: effect of population = -1299.4, p = 0.054; R ^2^ = 0.102). For a more thorough treatment of this population difference, see Atwell *et al.* (in review). This population-only model performed better than all other tested models (Akaike's Information Criterion = 670.8; AIC for all other models > 671.32).

## Discussion

Previous studies have related migratory behavior in birds and fish to allelic variation in *CLOCK* and *ADCYAP1*. This study examined whether variation in these genes also relates to migratory behavior in the avian genus *Junco*. At the level of populations, we found that longer *CLOCK* alleles were associated with longer migratory distance within two sub-specific groups, *J. h. oreganus* and *J. h. hyemalis*. *CLOCK* was not, however, significantly associated with migratory distance across the genus as a whole or with individual intensity of migratory restlessness. In the case of *ADCYAP1*, longer alleles were not associated with migratory distance among populations within subspecies groups or across the genus. However, longer alleles were associated with higher levels of individual migratory restlessness in one of two populations studied. Together these findings suggest that allelic variation in both *CLOCK* and *ADCYAP1* may contribute to differences in migratory behavior at some levels of analysis (within-subspecies or within-populations) but not reliably across the genus.

The lack of consistent relationships between molecular genetic variation and migratory variation suggested by our data are overall consistent with previous research on both *CLOCK* and *ADCYAP1*, as these genes are related to migratory and breeding behavior in only some of the species investigated. Within birds, *CLOCK* is associated with migratory and breeding behavior in blue tits ^19^, but not black caps, bluethroats, great tits, or swallows ^18, 24, 25, 59^. Similarly, a relationship between *ADCYAP1* and migratory behavior has been found only in blackcaps ^24^, but has yet to be reported in other species. Despite these conflicting results, allelic variation in each of these genes is potentially consistent with a direct relationship with migratory behavior (see below), suggesting that these genes may indeed be important to the evolution of migratory and breeding behavior.

There are at least three reasons why the associations reported here might differ according to which populations or species were investigated: 1) variation in other, related genes (i.e., genetic background); 2) variation in the degree of linkage between the allelic variation we measured and functional genetic differences; or 3) variation in the environment under which the association was sought. These explanations have been suggested for other genes with variable patterns of association with phenotype (e.g., Korsten *et al.* ^32^). We consider how each of these three explanations for variation in relationship applies to migratory behavior and *CLOCK* or *ADCYAP1*, and we suggest that population differences in genetic background are the most likely explanation. If this is indeed the case, then future studies of candidate genes will benefit from expanding the number of target genes examined to include related genes that may contribute to differences in genetic background.

### Mechanisms relating allelic variation to migratory phenotype

*CLOCK* alleles differ by multiples of three base pairs ( Table 4), consistent with previous results in other avian species (e.g., Johnsen *et al.* ^18^, Steinmeyer *et al.* ^43^) and with the location of the repeat within the coding region of *CLOCK* ^43^. It has previously been suggested that presence in the coding region might make these polymorphisms functional ^18^, particularly because the repeat is responsible for a polyglutamine tract expansion in a region known to control the rate at which the *CLOCK* protein activates the downstream circadian pathway ^60^. Polyglutamine expansions control the activity of many genes (e.g., Chamberlain *et al.* ^61^). Thus, variation in allele length could play a direct role in modifying migratory behavior by altering the threshold day length signal to activate or suspend migratory behavior, or by modifying its downstream effects.

The *ADCYAP1* alleles varied primarily by 2 base pairs ( Table 3), consistent with previous results and the fact that this microsatellite polymorphism falls within the 3´ untranslated region (UTR) of the *ADCYAP1* gene ^24, 43^. The mechanism by which this variation may relate to behavioral differences remains unknown. Di-nucleotide repeats in the regulatory regions of genes, such as 3´ UTR, can modify the expression of a gene ^62^. This polymorphism may simply be linked with another causative mutation in the coding region ^24^. The amino acid code of *ADCYAP1*, however, is 97% conserved from humans to chickens ^28^, making 3´ UTR based variation in expression far more likely than a polymorphic variation in protein sequence. Studies of *in vivo* expression of various alleles in multiple tissues would be necessary to confirm a role for this mutation in modifying the expression of *ADCYAP1* in living animals.

### Role of variation in genetic background

Migratory behavior is a complex phenotype consisting of changes to movement, orientation, feeding, fattening, and sleep patterns ^53^, and therefore may be modulated by many genes. The fact that *CLOCK* and *ADCYAP1* are related to migration in several systems suggests that they may be some of the key regulators. However, the large component of migratory behavior that is not explained by these genes may be related to other genes in related pathways. Changes in these other, related genes may thus reduce or modify the effect of *CLOCK* and *ADCYAP1* on migratory behavior, and explain the pattern of relationships identified in both previous studies ^18, 24, 25, 59^ (described above) and this work. Similar patterns have been found for other complex traits such as response to stress, including in experimental evolution of yeast ^63^. Despite similar repeated phenotypes, independently evolved lineages did not duplicate the same transcriptional (and therefore genetic) responses to a novel stressor ^63^. This suggests that divergent *Junco* populations may have utilized unique genetic mechanisms to achieve the same phenotypic end, thus masking the role of any single candidate gene across the genus.

Differences in genetic background may explain the lack of genus-wide patterns of relationship between migratory phenotype and *CLOCK* or *ADCYAP1* and the pattern of relationship found in previous studies. If other, as yet unidentified, genes also contribute to divergence in migratory and breeding behavior, then the relationships identified between phenotype and genotype would be limited to the groups or species in which *CLOCK* or *ADCYAP1* are responsible for the divergence. In contrast, those species and populations that do not demonstrate a relationship between migratory phenotype and *CLOCK* or *ADCYAP1* may have diverged due to changes in other genes. These genes might be involved in the circadian pathway, such as *BMAL1* or *Period 2* ^64^; the sensing of photoperiod changes, such as thyroid hormone deiodinases ^65^ or vertebrate ancient opsin ^66^; or transcriptional regulation of these systems ^67^.

Differences in genetic background may also explain the details of the relationships found between migratory phenotype and *CLOCK* or *ADCYAP1*. The Oregon and slate-colored subspecies groups both demonstrate a positive relationship between *CLOCK* allele length and migratory behavior. Importantly, the population mean *CLOCK* allele lengths are not different between the subspecies groups, and all of the slate-colored juncos migrate longer distances than the Oregon juncos. Similarly, allele length of *ADCYAP1* predicts migratory restlessness in the altitudinal migrant Laguna Mountain population, but not in the sedentary San Diego population despite the fact that both populations exhibit variation at the locus and in migratory restlessness. While sedentary populations are known to exhibit seasonal migratory restlessness ^68^, it is possible that changes in other genes inhibit migration in this sedentary population sufficiently to overwhelm the variation in restlessness contributed by *ADCYAP1*. Together, these results suggest that variation in other genes across the genus account for some of the shifts in migratory phenotype, but that *CLOCK* and *ADCYAP1* may still play important roles in further modification of migratory behavior.

### The role of linkage

An alternative explanation for the finding that allele length of both *CLOCK* and *ADCYAP1* were related to migratory behavior at only some levels (within subspecies or population) of analysis is that the alleles we assessed are not functional, but are instead genetically linked to functional differences. If so, this linkage may be distorted in some populations due to recombination or mutation. For those populations, the alleles we measured would no longer be informative as indicators of migratory behavior. The presence of the relationship in multiple independently derived lineages could be explained by independent events that link the allele at the causal locus to the same microsatellite alleles. For example, in great tits ( *Parus major*), only one of four populations studied, demonstrated a relationship between a single nucleotide polymorphism (SNP) in dopamine receptor 4 ( *DRD4*) and exploratory behavior ^32^, perhaps due to the breaking of a genetic linkage ^69^.

In the case of *CLOCK* and *ADCYAP1*, however, several pieces of evidence point against disrupted linkage as the source of variation in the genotype-phenotype relationship. Similarly to previous studies that found a significant relationship between *CLOCK* or *ADCYAP1* and migratory behavior (described above), the alleles associated with increased migratory behavior in this study were always longer. If linkage were responsible for the association, there is no reason to predict that longer alleles would repeatedly be linked to increased migratory propensity. This does not, by itself, rule out linkage–it is possible that this directional pattern is coincidental and will be reversed in another species yet to be studied. In addition, unlike the length polymorphisms studied in *CLOCK*, the SNP in *DRD4* related to exploratory behavior is a synonymous mutation ^69^–that is, it causes no change in the protein that is encoded by the gene. Similarly, the variation in *ADCYAP1* is predicted to affect expression ^43^, while the SNP in *DRD4* is unlikely to do so (but see Duan *et al.* ^70^). Given the existence of a known mechanism that could relate allelic variation in *CLOCK* and *ADCYAP1* to functional consequences (detailed above), disrupted linkage seems less likely to account for variable strength of association than other possible causes.

### The role of environmental factors

A final potential explanation for the lack of a consistent relationship between allelic variation and migratory phenotype is that the roles of *CLOCK* and *ADCYAP1* are modulated by the different environments that populations and subspecies experience. In three-spine sticklebacks ( *Gasterosteus aculeatus*), for example, environmental variability in the presence of predators masks genetic variation related to several behavioral traits ^71^. Similarly, many environmental cues, such as temperature and precipitation, modulate migratory behavior ^9^; such environmental modulators may account for some of the differences between junco populations and may mask the effects of variation in *CLOCK* and *ADCYAP1*.

Environmental differences do not, however, appear to be consistent with the pattern of relationships found between migratory phenotype and *CLOCK* or *ADCYAP1*. First, the positive relationship between individual variation in migratory restlessness and length of *ADCYAP1* was found in only one of two tested populations (Laguna Mountain), despite the fact that they were tested in an identical common garden experiment ^48^, and winter in the same environment ^41^. Second, the within subspecies pattern seen between *CLOCK* allele length and migratory distance is seen in both Oregon and slate-colored juncos, and these two subspecies differ in the amount of environmental divergence between populations. The sampled Oregon populations breed in divergent climates and winter in similar environments, while the slate-colored populations all breed in a similar environments, but migrate different distances to divergent wintering grounds ^34, 41^. Together, the findings in *CLOCK* and *ADCYAP1* suggest that environmental differences between populations, while potentially important, are not sufficient to explain the pattern of associations that we found.

## Conclusions

We found no evidence for a predictable relationship between migratory behavior and the lengths of *CLOCK* or *ADCYAP1* alleles across the genus *Junco*. However, we found some support for a role of *CLOCK* allele length within subspecies groups, and *ADCYAP1* allele length within a population, in modifying migratory behavior. The lack of consistent detectable associations between allelic variation and behavioral variation among populations and individuals as reported in our study, adds an important layer in further understanding the potential roles of candidate genes in complex ecological and evolutionary processes. To explore this layer further, future sequencing projects may wish to similarly consider both between-group and within-group variability in genotype-phenotype relationships when conducting analyses of other candidate genes.

Focusing on variation in a small number of genes with predicted functional significance holds the potential to identify major functional hubs in complex phenotypes across the animal kingdom. The redundancy of the genome and complexity of functional genetic, regulatory, and signaling networks, however, means that over evolutionary time scales, candidate loci may gain or lose functional significance in the presence of an alternative environment, linkage, or genetic background. Our results highlight the fact that different mechanisms may contribute to variation in complex phenotypes when examining populations or species. This does not mean that candidate gene approaches should be abandoned altogether. Rather, with the increasing availability of next-generation sequencing, it may become more efficient to expand the scope of candidate gene sequencing projects to include other genes in the same pathway, as this breadth may provide insight into other, as yet unidentified, genetic mechanisms.

## Figures and Tables

**Supplementary Table 1.** Individual variation in migratory restlessness and allele length. Allele calls for both CLOCK and ADCYAP1 are provided for each individual. Migratory restlessness score provides a cumulative score of migratory behavior over the sampling period (March 3 to August 1). See Methods for details.

Sample ID	Population	CLOCK1	CLOCK2	ADCYAP1	ADCYAP2	Migratory restlessness score
5203	ORJU-LM	273	276	163	165	3660
5226	ORJU-LM	276	276	163	164	3785
5244	ORJU-LM	276	276	159	163	6116
5245	ORJU-LM	276	276	159	163	481.8
5246	ORJU-LM	267	276	161	164	604.2
5249	ORJU-LM	276	276	161	163	6020
5250	ORJU-LM	276	276	163	163	126.3
5251	ORJU-LM	270	276	161	161	596.5
5253	ORJU-LM	276	276	159	161	333.6
5256	ORJU-LM	276	276	165	165	8648
5260	ORJU-LM	276	276	161	165	4477
5263	ORJU-LM	276	276	161	165	17.5
5437	ORJU-LM	276	276			3629
5492	ORJU-LM	276	276	159	163	658.2
5504	ORJU-LM	273	276	161	163	2384
5562	ORJU-LM	276	276	159	161	1230
5586	ORJU-LM	276	276	159	165	3851
5592	ORJU-LM	270	276	159	163	5412
5103	ORJU-SD	270	276	161	165	1963
5142	ORJU-SD	276	276	163	163	1159
5231	ORJU-SD	270	276	161	167	2433
5232	ORJU-SD	270	276	161	161	10.35
5404	ORJU-SD	270	276	161	167	1616
5427	ORJU-SD	270	276	161	163	915.9
5489	ORJU-SD	276	276	157	157	1306
5518	ORJU-SD	276	276	161	163	3742
5519	ORJU-SD	276	276	163	167	2337
5544	ORJU-SD	270	276	161	163	724.2
5545	ORJU-SD	276	276	159	161	2211
5550	ORJU-SD	270	276	159	161	458.3
5556	ORJU-SD	270	276	163	167	32.67
5557	ORJU-SD	270	276	161	163	473.7
5565	ORJU-SD	276	276	159	161	683.7
5566	ORJU-SD	276	276	157	157	788
5568	ORJU-SD	270	276	157	163	3644
5569	ORJU-SD	276	276	157	161	3766
