# Supplementary material for: Variation in candidate genes CLOCK and ADCYAP1 does not consistently predict differences in migratory behavior in the songbird genus Junco
Source: F1000Res. 2013 Apr 22;2:115. [Version 1] doi: 10.12688/f1000research.2-115.v1 (PMC3907158; doi:10.12688/f1000research.2-115.v1)
Supplement: Peer review history

# Peer review: Referee response for version 1

**Published:** 2013-05-13

**Authors:** Roi Dor

**Affiliations:** 1 Ecology & Evolutionary Biology, University of Colorado Boulder, Boulder, CO, USA

Peterson and co-authors have investigated the association between genotypes of two candidate genes ( *Clock* and *ADCYAP1*) and migratory behaviour in the avian genus *Junco*. They examined this association among subspecies, populations and individuals. Previous studies have found contradicting results regarding the association between phenotypic variation and genotype of these genes; therefore, more similar studies are required in order to determine the generality of this relationship and the role of these genes.

The authors have sampled and analysed a large number of individuals and populations and analysed the data adequately. Unfortunately, the results are mostly negative and prevent from reaching clear-cut conclusions regarding *Clock* or *ADCYAP1* and migratory propensity. However, studying the relationship between genotypes and phenotypes on several levels (subspecies, population, and individuals) enabled performing interesting comparisons and detecting some positive patterns.

The authors have properly discussed the results and potential explanations and mechanisms for the differences found among the levels examined.

I agree with the authors that correlating genomes with phenotypic variation may be the next step to identify potential candidate genes that may have a role in complicated behaviours such as migration.

Despite the mostly negative results, I believe that this nicely-written paper provides a fine contribution to the literature.One technical comment – it would be interesting to learn the nature of the *ADCYAP1* alleles that are just one base-pair apart.

I have read this submission. I believe that I have an appropriate level of expertise to confirm that it is of an acceptable scientific standard.

# Peer review: Referee response for version 1

**Published:** 2013-05-08

**Authors:** Francisco Pulido

**Affiliations:** 1 Vertebrate Biology and Conservation, Universidad Complutense de Madrid, Madrid, Spain

I have read this submission. I believe that I have an appropriate level of expertise to confirm that it is of an acceptable scientific standard.

# Peer review: Referee response for version 1

**Published:** 2013-04-30

**Authors:** Miriam Liedvogel

**Affiliations:** 1 Centre for Animal Movement Research, Lund University, Lund, Sweden

Peterson and coworkers investigate phenotypic correlates of genetic variation in two species of the bird genus *Junco* at the level of individuals, populations and species across taxa, which is a valuable contribution to our understanding of the genetic basis of the complex behavioural phenotype of migration. The methodological approach the authors have chosen is a candidate gene approach at two loci ( *Clock* and *ADCYAP1*), which have previously been analysed in the context of their putative involvement in shaping migratory phenotypes in different taxa. The study is a direct follow up from the study by Müller *et al*. 2010, and a very welcomed attempt to repeat previous findings in another system to obtain a better understanding of associations between genetic variation and behaviour, as well as to assess their generality. This is much needed since associations between polymorphisms in candidate genes and behavioural traits have often proven to be difficult to repeat across populations or species, and replication is indeed a valuable tool to infer the generality of association patterns.

The difficulty and my primary concern with the study as well as the precursor study is that candidate genes (defined as genes that have been studied in one (model) organisms and there have been found to influence the expression of one focal phenotype) for migratory traits are probably not know to date, as none of the model organisms show a migratory phenotype. The genes in focus here are candidate genes for candidate traits, probably involved in shaping the migratory phenotype (i.e. the circadian phenotype involved in timing of migration). Thus, conclusions drawn upon these data based on correlative evidence should be evaluated with great caution. 

This study is useful in the way that it adds data to the pool of studies that have explored candidate genes of candidate traits for the migratory phenotype, which are difficult to interpret and don’t allow drawing any clear conclusions upon. The study is well presented and the additional data confirm the apparent inconsistency and the difficulty to interpret these data, probably highlighting the weakness of the candidate gene approach in this field of migratory research, and I support their publication. With candidate gene studies per se we do not expect to get huge effects, and in the area of migration genetics, I suggest that future research should avoid using a candidate gene approach, as this is unlikely to yield clear results - at least unless clear candidate genes for the migratory phenotype have been identified *de novo*.

I have read this submission. I believe that I have an appropriate level of expertise to confirm that it is of an acceptable scientific standard.
